# Research Progress on the Role of *Lactobacillus reuteri* in Irritable Bowel Syndrome: A Review

**DOI:** 10.3390/biology15100808

**Published:** 2026-05-20

**Authors:** Jinyi Zhen, Zhengrong Zhou, Ruiqi Zhu, Meiqian Kuang, Pan Huang

**Affiliations:** School of Medicine, Jiangsu University, Zhenjiang 212013, China; 3210910005@stmail.ujs.edu.cn (J.Z.); zrzhou@ujs.edu.cn (Z.Z.); 3221401134@stmail.ujs.edu.cn (R.Z.); 1000005730@ujs.edu.cn (M.K.)

**Keywords:** *Lactobacillus reuteri*, IBS, intestinal barrier, immunomodulation, gut–brain axis

## Abstract

Irritable bowel syndrome (IBS) is a multifactorial functional intestinal disorder whose pathogenesis involves intestinal microbiota imbalance, mucosal barrier impairment, immune–inflammatory responses, and dysregulation of the gut–brain axis. *Lactobacillus reuteri* has been shown to modulate the gut microbiota, produce antimicrobial metabolites, strengthen the intestinal mucosal barrier, suppress inflammatory responses, and participate in gut–brain axis regulation, thereby potentially contributing to the maintenance of intestinal homeostasis and the alleviation of IBS-related symptoms. These findings indicate that *L. reuteri* holds potential therapeutic value in the prevention and treatment of IBS. However, the majority of supporting evidence derives from animal experiments, in vitro studies, or indirect sources, and its precise mechanisms of action and clinical efficacy warrant further investigation.

## Simple Summary

Irritable bowel syndrome (IBS) is a common functional gastrointestinal disorder with a multifactorial and incompletely understood etiology, and currently available treatments show variable efficacy. Alterations in the gut microbiota are thought to contribute to IBS pathophysiology, and certain probiotic strains, such as *Lactobacillus reuteri*, have attracted interest as potential therapeutic candidates. This review examines the current evidence on the possible mechanisms by which *L. reuteri* may influence IBS, including modulation of the gut microbiota, enhancement of intestinal barrier function, attenuation of inflammation, and regulation of the gut–brain axis. While mechanistic findings from preclinical studies provide supportive evidence, robust confirmation from high-quality clinical trials remains limited, and further research is needed to validate its clinical efficacy and to guide personalized probiotic therapy. This review aims to provide a foundation for future investigation and to inform the evidence-based translation of *L. reuteri* into IBS management.

## 1. Introduction

Irritable bowel syndrome (IBS) is a functional intestinal disorder with higher prevalence among young and middle-aged adults (aged 18–59 years), characterized by recurrent abdominal pain often accompanied by altered bowel habits. The pathophysiological mechanisms of IBS have not been fully elucidated. It is currently believed to result from abnormal gut–brain interaction caused by the combined effects of multiple factors [1]. The clinical management of IBS primarily adheres to the principles of comprehensive management and individualized treatment. Commonly used medications include antispasmodics, antidiarrheals, laxatives, and intestinal microbiota preparations. Given the complex etiology of IBS, single therapeutic approaches often fail to address all pathological mechanisms. As a chronic condition, current treatments or short-term dietary modifications frequently yield suboptimal long-term outcomes. Clinical studies have demonstrated that probiotics can alleviate abdominal distension, abdominal pain, diarrhea, and overall symptoms in IBS patients, suggesting their promising therapeutic potential.

Individuals with IBS frequently present with altered gut microbial communities, typically characterized by reduced microbial diversity relative to healthy controls. However, the specific compositional shifts reported across studies are marked by considerable heterogeneity and vary substantially according to IBS subtype. For example, diarrhea-predominant irritable bowel syndrome (IBS-D) patients tend to show increased Firmicutes and decreased Bacteroidetes, whereas constipation-predominant irritable bowel syndrome (IBS-C) patients more commonly exhibit decreased Firmicutes and Actinobacteria with increased Verrucomicrobiota and Proteobacteria. In IBS-C, beneficial taxa such as *Bifidobacterium* and *Lactobacillus* are frequently reduced, while pro-inflammatory microbes such as Enterobacteriaceae are enriched [2,3,4]. These changes may lead to impaired intestinal barrier function, abnormal immune responses, and disrupted neural signaling, thereby exacerbating IBS symptoms [5]. *Lactobacillus reuteri* (*L. reuteri*) belongs to the Firmicutes phylum, Bacilli class, Lactobacillales order, Lactobacillaceae family, and *Lactobacillus* genus. It is a hydrogen peroxidase-negative, Gram-positive, non-motile, non-spore-forming, obligate heterotrophic fermentative bacterium primarily colonizing the gastrointestinal tract. Studies have shown a significant reduction in *L. reuteri* abundance in the intestines of IBS patients [6,7]. *L. reuteri* may contribute to regulating the gut microbiota, eliminating infections, and alleviating clinical symptoms of diseases such as IBS, antibiotic-associated diarrhea, inflammatory bowel disease, and chronic constipation [8]. While prior reviews have examined the potential roles and therapeutic effects of probiotics in intestinal disorders such as inflammatory bowel disease (IBD), ulcerative colitis (UC), and IBS, *L. reuteri* has typically been mentioned only as one among many probiotic candidates. This review aims to synthesize the current evidence on the regulatory effects of *L. reuteri* in IBS across four interconnected mechanistic dimensions: modulation of the gut microbiota, restoration of barrier integrity, immune regulation, and gut–brain axis signaling. Where the available data permit, we further attempt to delineate the functional characteristics of different *L. reuteri* strains in the context of distinct IBS subtypes. Finally, the potential clinical application value of *L. reuteri* intervention for IBS was preliminarily evaluated.

## 2. Review Methodology and Search Strategy

PubMed and Web of Science were systematically searched from inception to May 2026 using combined terms for the probiotic (“*Lactobacillus reuteri*” OR “*Limosilactobacillus reuteri*” OR “*L. reuteri*”) and the condition (“irritable bowel syndrome” OR “IBS” OR “irritable colon” OR “functional gastrointestinal disorder”). Eligible studies were original research articles, reviews, or meta-analyses that focused on *L. reuteri* (any strain) in the context of IBS pathophysiology, symptomatology, or treatment, provided mechanistic insights, and were published in English. Conference abstracts, case reports and editorials without separate analysis were excluded. Multiple reviewers independently screened titles and abstracts, and subsequently full text articles, for eligibility, with disagreements resolved by consensus. Consistent with the narrative nature of this review, formal quality assessment or risk of bias scoring was not conducted; however, study limitations are critically discussed where relevant. A total of 72 articles were included in the final synthesis.

## 3. The Metabolites of *L. reuteri* Exhibit Antibacterial Activity

As shown in Table 1, *L. reuteri* metabolizes glycerol to produce reuterin, a complex molecular mixture primarily composed of 3-hydroxypropionaldehyde (3-HPA) and its spontaneously formed dimers, hydrates, and acrolein. These compounds exert antibacterial effects on Gram-positive bacteria, Gram-negative bacteria, yeasts, molds, and protozoa by inducing oxidative stress, disrupting cell membranes, and causing DNA damage [9,10]. Schaefer et al. discovered that dehydration of reuterin molecules generates the highly toxic compound acrolein, which modifies thiol proteins and functional groups in small molecules, leading to redox imbalance and metabolic disturbances, thereby triggering cellular oxidative stress responses and inhibiting various microorganisms [11]. Additionally, molecular docking simulations conducted by Purnawita et al. demonstrated that the active component 3-HPA of reuterin inhibits the activity of antioxidant enzymes such as the catalase family, further increasing intracellular reactive oxygen species (ROS) levels and inducing oxidative stress, ultimately resulting in cell death [12]. In addition to directly killing bacteria, studies have found that reuterin can induce loss of membrane integrity in *Clostridioides difficile* and increase DNA damage by 7.6-fold in TUNEL assays, confirming that reuterin also generates ROS leading to impaired metabolism in *C. difficile*. Reuterin further enhances the sensitivity of *C. difficile* to vancomycin and metronidazole [13]. The extracellular polysaccharide (EPS) layer produced by *L. reuteri* not only physically impedes the adhesion of pathogens such as *Salmonella typhi* but also concentrates Reuterin, creating a high-concentration Reuterin environment locally, thereby more effectively inhibiting the growth of competing microorganisms [14].

*L. reuteri* can regulate microbial community composition by secreting short-chain fatty acids (SCFAs) such as acetic acid, along with other organic acids like lactic acid. Through the fermentation of glucose to produce organic acids such as acetic acid and lactic acid, *L. reuteri* I5007 establishes an acidic microenvironment conducive to the growth of bacteria like *Lactobacillus* [15,16]. The acidic environment disrupts the charge balance of certain microbial cell membranes, leading to increased membrane permeability, and also affects microbial protein synthesis and enzyme activity. Consequently, it inhibits the colonization of harmful bacteria such as Proteobacteria and Fusobacteria, while promoting the colonization of beneficial bacteria like Bacteroidetes and Bacillota that produce butyrate, thereby elevating butyrate levels in the gut and balancing the intestinal microbiota [17,18]. *Proteus mirabilis*, a representative enteric pathogen within the phylum Proteobacteria, can suppress IL-18 expression in intestinal epithelial cells, downregulate mucin production, and aggravate UC [19]. These findings suggest that *L. reuteri* may reinforce the intestinal mucosal barrier in part by suppressing the expansion of Proteobacteria, including *P. mirabilis*. Members of the Bacteroidetes phylum, widely recognized as keystone taxa within the gut microbiota, are essential for maintaining gastrointestinal eubiosis. Their metabolic products, including SCFAs and secondary bile acids, attenuate intestinal inflammation, while capsular polysaccharides and sphingolipids contribute to mucosal immune regulation and the promotion of intestinal homeostasis [20]. These beneficial effects suggest that *L. reuteri* may, in part, alleviate intestinal mucosal inflammation by promoting the enrichment of beneficial Bacteroidetes. Additionally, *L. reuteri* competes with other microorganisms for nutrients and colonization space, limiting the growth of harmful bacteria [21,22,23].

## 4. *L. reuteri* Promotes Mucosal Epithelial Injury Repair and Strengthens Intestinal Mucosal Barrier

The intestinal mucosal barrier (IMB) is the structural and functional complex that prevents harmful substances (e.g., bacteria, toxins) from penetrating the intestinal mucosa into other tissues and the circulatory system. It consists of four components: mechanical barrier, chemical barrier, immune barrier, and biological barrier [24]. Increased small intestinal permeability is observed in 37–62% of patients with diarrhea-type IBS (IBS-D), with a positive correlation between barrier dysfunction and symptoms such as abdominal pain and intestinal motility alterations [25]. Silvia Cruchet and colleagues carried out a randomized, double-blind, placebo-controlled clinical study involving 140 adults aged between 18 and 65 years who were diagnosed with IBS based on the Rome IV criteria, comprising 23 cases of IBS-C, 33 cases of IBS-D, and 84 cases of IBS-M. After 2 weeks of washout, subjects were randomized to receive either 2 × 10^8^ colony-forming units (CFUs) of *L. reuteri* DSM 17938 combined with *L. reuteri* ATCC PTA 6475 plus standard of care or placebo plus standard of care for 14 weeks, followed by a post-intervention period of 2 additional weeks. Changes in gastrointestinal symptoms (as measured with the GSRS-IBS), stool pattern (as measured with the Bristol scale), quality of life, depression and anxiety, frequency of adverse events, and fecal calprotectin concentrations were evaluated. In experimental studies, the *L. reuteri* ATCC PTA 6475 and *L. reuteri* DSM 17938 strains have exhibited beneficial effects on the intestinal barrier. The combined administration of these two strains has been shown to improve stool consistency and decrease fecal calprotectin levels in patients with moderate-to-severe IBS-D, while also significantly ameliorating symptoms such as abdominal pain, pain relief associated with defecation, and abdominal bloating in patients with IBS-C [26]. In a 4-month, double-blind, randomized controlled trial, 55 children aged 4–18 years diagnosed with functional abdominal pain (FAP) or IBS were randomized to receive *L. reuteri* DSM 17938 (10^8^ CFU/day) or placebo. The probiotic group had significantly more pain-free days than the placebo group, and abdominal pain severity was significantly lower at months 2 and 4 [27]. When used as adjuvant therapy for *Helicobacter pylori* (*H. pylori*), this bacterial strain combination significantly alleviates gastrointestinal symptoms such as abdominal pain, bloating, and diarrhea, while improving intestinal barrier function [28].

The intestinal mucosal mechanical barrier is composed of epithelial cells lined with adherent junctions (AJs) and tight junctions (TJs) that connect the cells [29]. *L. reuteri* can directly and indirectly affect the mechanical barrier of the intestinal mucosa. The abundant lipoteichoic acid (LTA) and mucin-binding protein (Mub) adhesins present in *L. reuteri* 1063 extracellularly promote its adhesion to ileal mucus and epithelial cells, and after colonizing the host gastrointestinal tract, form biofilms that directly reinforce the intestinal mucosal barrier, effectively reducing the extraintestinal dissemination of commensal bacteria [30]. Studies in autism spectrum disorder (ASD) rat models have revealed that *L. reuteri* upregulates the expression of tight junction proteins (zonula occludens-1, ZO-1) and occludin to maintain intestinal barrier integrity and reduce intestinal permeability, though the underlying mechanisms require further investigation [31]. Notably, the phylogenetically related species *Lacticaseibacillus casei* enhances ZO-1 expression and strengthens intestinal barrier integrity through the activation of protein kinase C (PKC) downstream of Toll-like receptor 2 (TLR2). Given the close evolutionary relationship between these lactobacilli, it is plausible that *L. reuteri* employs a similar TLR2–PKC signaling axis to reinforce tight junction assembly [32].

In animal and cell-based models, *L. reuteri* has been shown to promote mucin secretion, increase mucus layer thickness, facilitate intestinal mucosal development, and improve intestinal morphological structure. These findings suggest a potential role in alleviating damage to the mechanical barrier. The *L. reuteri* LR1 strain can mitigate the increased permeability of intestinal epithelial cells (IPEC-1 cells) induced by enterotoxigenic E. coli K88 and reduces Escherichia coli adhesion and invasion in IPEC-1 cells [33]. Additionally, *L. reuteri* R2LC and *L. reuteri* 4659 can increase the number of ileal goblet cells in piglets to enhance mucus secretion, increase the thickness of the colonic mucus layer, and reduce contact between intestinal bacteria and intestinal epithelial cells [34].

*L. reuteri* D8 can induce intestinal stem cells (ISCs) to differentiate into Paneth cells (PCs), which secrete various signaling molecules such as epidermal growth factor (EGF), transforming growth factor-β (TGF-β), Wnt3, and Notch ligand DLL4 (Delta-like ligand 4), thereby promoting ISC proliferation [35]. In a mouse intestinal inflammatory injury model induced by tumor necrosis factor (TNF), the addition of *L. reuteri* D8 maintained PC counts and lysozyme expression at normal levels, thereby enhancing the chemical barrier of the intestinal mucosa [36]. In a broiler model with intestinal mucosal damage, *L. reuteri* was found to induce the expression of R-spondins, activate and maintain the Wnt/β-catenin signaling pathway, stimulate ISC expansion, inhibit intestinal cell apoptosis and inflammatory responses, and contribute to intestinal mucosal repair [37]. The metabolic product 3-HPA from viable *L. reuteri* can stimulate lamina propria lymphocytes (LPLs), prompting them to secrete interleukin-22 (IL-22) via the aryl hydrocarbon receptor (AHR), thereby inducing phosphorylation of signal transducer and activator of transcription 3 (STAT3), accelerating intestinal epithelial proliferation, restoring damaged intestinal mucosa, and preserving the morphology of intestinal organoids as well as normal epithelial proliferation [38,39]. In addition, in a mouse intestinal epithelial cell model, *L. reuteri* I5007 was found to upregulate the expression of *miR-196a* in MODE-K intestinal epithelial cells, which in turn targeted and downregulated the *Programmed cell death 4 (PDCD4)* gene. This led to reduced expression of the downstream protein Cysteinyl aspartate specific proteinase (Caspase-3), thereby inhibiting LPS-induced inflammatory responses and apoptosis in intestinal epithelial cells and protecting the intestinal epithelial barrier [40].

## 5. *L. reuteri* Modulates Intestinal Immunity and Exerts Anti-Inflammatory Effects

Low-grade inflammation and immune-neural activation are present in IBS. *L. reuteri* has been demonstrated to modulate intestinal immunity through multiple pathways, inhibiting the occurrence and progression of inflammation. Strains including *L. reuteri* DSM17938, ATCC PTA4659, ATCC PTA 5289, and ATCC PTA 6475 are capable of suppressing LPS-induced KC/GRO expression in rat intestinal mucosal cells, diminishing interferon-gamma (IFN-γ) levels, inhibiting the recruitment and activation of neutrophils, and thereby exerting anti-inflammatory effects [41,42]. Additionally, *L. reuteri* regulates the NF-κB signaling pathway, which is closely associated with inflammation, through multiple mechanisms. In ASD rat models, *L. reuteri* significantly reduced colonic p-NF-κB expression levels [31]. In weaned pig ileal models, *L. reuteri* ZLR003 was found to markedly upregulate glutathione peroxidase 2 (GSH-Px2) expression, enhancing intestinal antioxidant capacity and barrier function in weaned piglets while inhibiting the NF-κB signaling pathway [43,44]. The 3-HPA contained in the membrane vesicles derived from *L. reuteri* significantly increases anti-inflammatory factors such as interleukin-10 (IL-10) in mouse tongue tissues while reducing pro-inflammatory factors including TNF-α, interleukin-1β (IL-1β), and interleukin-6 (IL-6). Additionally, it stabilizes the mitochondrial membrane potential affected by LPS by modulating the activity of respiratory complexes CI and CII, thereby decreasing mitochondrial and intracellular ROS production and inhibiting the NF-κB signaling pathway [45]. The *L. reuteri* BNCC 186135-galactooligosaccharide synbiotic can metabolically produce N-acetyl-D-glucosamine, increase the abundance of *Bifidobacterium acidifaciens*, promote the synthesis of pentadecanoic acid (C15:0) by B. acidifaciens, activate fatty acid translocase (FATP4) in colitis model mice, and inhibit the phosphorylation of NF-κB p65 [46].

Histamine produced by *L. reuteri* ATCC PTA 6475 increases intracellular cAMP production in mucosal cells through H2 receptor signaling, blocks the activation of the MEK/ERK MAPK signaling pathway, and exerts anti-inflammatory effects by inhibiting AP-1 translocation to the nucleus, thereby suppressing TNF gene transcription [47,48]. In a murine model of tumor immune checkpoint blockade (ICB)-associated enteritis induced by vancomycin, *L. reuteri* ATCC PTA 6475 alleviated ICB-associated enteritis by reducing intestinal ILC3s lymphocyte populations, suggesting that *L. reuteri* may exert a mitigating effect on inflammation induced by IBS through modulation of intestinal lymphocyte subsets [49].

Patients with constipation-type IBS (IBS-C) exhibit reduced levels of propionate and butyrate in the intestines, whereas IBS-D patients demonstrate increased butyrate levels [50]. Targeted metabolomics analysis in ASD rat models revealed that *L. reuteri* significantly reduced propionic acid levels and increased butyric acid levels in feces [31]. Butyrate can stimulate macrophages to produce nitric oxide (NO), which inhibits the release of pro-inflammatory cytokines by various immune cells. Additionally, NO can suppress visceral abnormal pain and colonic hyperpermeability in IBS rat models through central dopamine D2 receptors and opioid pathways, thereby ameliorating clinical symptoms of IBS [51]. Moreover, butyrate upregulates MUC2 gene expression and stimulates goblet cell mucin secretion, thereby thickening the mucus layer. It also acts synergistically with IL-1β to induce antimicrobial peptide production—exemplified by LL-37—thus enhancing the chemical defense of the mucosal barrier. In parallel, circulating butyrate can cross the blood–brain barrier or act on vagal afferent fibers, thereby modulating central nervous system activity through peripheral signals, suppressing excessive microglial activation, and attenuating neuroinflammation. Indirectly, butyrate modulates the synthesis of neurotransmitters such as serotonin and γ-aminobutyric acid (GABA), with consequent effects on mood and cognition. Furthermore, butyrate regulates the hypothalamic–pituitary–adrenal (HPA) axis, attenuating cortisol release and thereby shaping the host’s physiological stress response [52]. These observations further suggest that *L. reuteri* may exert protective effects in IBS in part through butyrate-mediated enhancement of the intestinal mucosal barrier and modulation of neurotransmitter synthesis.

## 6. *L. reuteri* Regulates Neuroendocrine Function via the Gut–Brain Axis

The “microbiome-gut-brain axis” constitutes a complex, bidirectional communication network that tightly links the microbiome, the gut itself, and the brain and nervous system. Patients with IBS often exhibit comorbid psychological abnormalities such as anxiety, depression, and neuroticism, and the adverse psychological states induced by IBS are significantly correlated with patients’ quality of life. Accumulating evidence, primarily from animal and cell-based models, suggests that *L. reuteri* may modulate neuroendocrine functions through multiple mechanisms, thereby potentially contributing to the alleviation of certain IBS-related symptoms.

### 6.1. L. reuteri Modulates Host Tryptophan Metabolism to Alleviate Depressive Symptoms

Patients with IBS-D exhibit significantly higher 5-HT concentrations compared to healthy individuals and IBS-C patients, with markedly reduced expression and activity of serotonin transporter (SERT) in the colonic mucosa of IBS-D patients [53,54]. In a randomized controlled trial, *L. reuteri* DSM 17938 was administered to 56 functional constipation (FC) patients for 105 days in a randomized, double-blind manner. The fasting blood samples were collected during the randomization visit (V1), at day 15 (induction period, V2), day 60 (intermediate evaluation, V3), and day 105 (V4) and the Constipaq questionnaire (the sum of Constipation Scoring System (CSS) and patient assessment constipation quality of life (PAC-QoL)) was administered. A group of healthy subjects was enrolled as controls (HC). The results revealed that *L. reuteri* DSM 17938 can significantly downregulate 5-HT levels in the rectal mucosa, relieve constipation symptoms, and improve the quality of life in patients with functional constipation [55]. Transcriptomic analysis revealed that *L. reuteri* 100-23 harbors the Glu/GABA reverse transporter proteins gadC1 and gadC2, as well as the codon for glutaminase subtype 3 (gls3), suggesting that *L. reuteri* can convert glutamate into GABA [56]. In male Cntnap4 gene knockout mouse models, it was demonstrated that *L. reuteri* ATCC PTA 6475 enhances GABA transmission in the basolateral amygdala (BLA) and ameliorates social deficits and fear memory processing impairments in mice [57,58]. In a mouse model of depression susceptibility induced by chronic social defeat stress (CSDS), *L. reuteri* 3 was found to significantly reduce kynurenine levels in both the blood and brain, thereby preventing depression-like behaviors caused by chronic social defeat stress [59]. *L. reuteri* I5007 metabolizes endogenous N-methyl-D-aspartic acid (NMDA) receptor antagonists, such as kynurenic acid (KYNA), 3-hydroxykynurenine, and ortho-aminobenzoic acid, which are derived from kynurenine/acetylated kynurenine. This process competes with the host’s kynurenine pathway, thereby lowering kynurenine levels and modulating T-cell responses that target the central nervous system [60]. Furthermore, KYNA exerts antioxidant effects by scavenging ROS and superoxide anions, as well as neuroprotective effects through the clearance of peripheral glutamate. These findings, derived primarily from non-IBS animal models, indirectly suggest that *L. reuteri* might alleviate psychological comorbidities such as depression in patients with IBS via the kynurenine pathway, but this requires direct validation in IBS-specific studies [61].

### 6.2. L. reuteri Promotes Host Oxytocin Secretion and Reduces Intestinal Sensitivity

Patients with IBS exhibit significantly higher levels of oxytocin receptor (OTR) in the colonic mucosa compared to healthy individuals, with variations observed across different IBS subtypes [62,63]. In a skin injury mouse model, administration of live *L. reuteri* ATCC PTA 6475 bacteria and their lysates was found to stimulate enteroendocrine cells in the intestine to produce secretin, thereby promoting the production and release of oxytocin (OT) by intestinal cells [64,65]. In an autism mouse model, *L. reuteri* ATCC PTA 6475 was demonstrated to exert OT-mediated effects on the vagus nerve, restoring socially induced synaptic plasticity in the ventral-dorsal tegmental nucleus of the midbrain and reversing social behavioral deficits in mice. However, this effect was not observed in OTR-deficient mice. Additionally, OT improved intestinal peristalsis, reduced scores in abdominal withdrawal reflex (AWR) experiments, and alleviated IBS symptoms [66,67]. OT can also activate Gq proteins coupled to oxytocin receptors in intestinal smooth muscle cells, thereby inhibiting gastrointestinal smooth muscle contraction by modulating the release of intestinal neurons and neurotransmitters, and mitigating stress-induced hypermotility of the colon [68]. However, in the IBS rat model, it was found that GABAergic neuron projections in the paraventricular nucleus of the hypothalamus (PVN) were significantly increased, inhibiting neuronal firing rates and reducing OT expression. This suggests that *L. reuteri* may exert a relief effect on IBS by regulating OT metabolic levels.

### 6.3. L. reuteri Inhibits the HPA Axis to Alleviate Stress Responses and Exerts Analgesic Effects

As a disorder characterized by abnormal gut–brain interaction, IBS is closely associated with stress stimuli. Both acute and chronic stress can induce or exacerbate symptoms in IBS patients. Imaging studies reveal abnormal activation patterns in the prefrontal cortex, insula, and anterior cingulate cortex of IBS patients, accompanied by diminished neural network capacity for visceral stimulus perception and modulation. The imbalance in sympathetic–parasympathetic nerve activity leads to intestinal motility rhythm disturbances and sensory amplification. Overactivation of the HPA axis results in sustained elevation of cortisol levels, heightened visceral pain sensitivity, and suppression of intestinal immune tolerance [69]. In an LPS-induced stress mouse model, *L. reuteri* was found to significantly reduce serum corticotropin-releasing hormone (CRH) and corticosterone levels, upregulate glucocorticoid receptor NR3C1, alleviate excessive activation of the HPA axis, mitigate stress responses, thereby improving stress-induced increased intestinal sensitivity, elevated inflammatory levels, and nociceptive responses. Furthermore, it suppressed NF-κB/NOX1 activation and improved barrier integrity, pointing to a potential dampening effect on HPA axis hyperactivity. This attenuation of the stress response may have favorable implications for IBS pathophysiology [31].

## 7. Limitations and Future Perspectives

**Figure 1:**
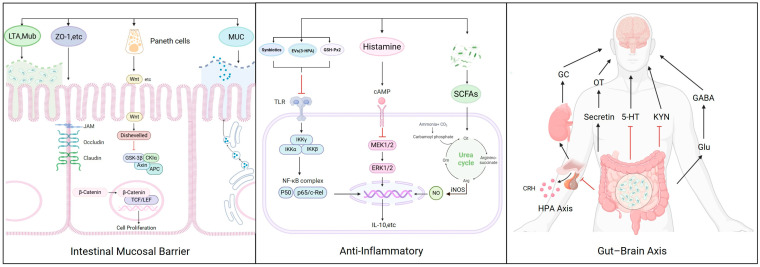
Multifaceted mechanisms of *L. reuteri* potentially relevant to IBS pathophysiology. Schematic overview of actions by which *L. reuteri* could influence IBS-related pathways, including gut microbiota modulation, barrier reinforcement, immunoregulation, and gut–brain axis signaling. The evidence for these mechanisms varies considerably. Several depicted pathways—particularly neuroendocrine and certain anti-inflammatory effects—have been established primarily in preclinical systems and await confirmation in human IBS. This figure should therefore be viewed as an integrative framework of potential interactions, not as a summary of equally validated therapeutic targets.

As summarized in Figure 1, *L. reuteri* may exert potentially protective effects in IBS through multiple proposed mechanisms; however, direct clinical evidence remains limited and many of these pathways have been demonstrated mainly in preclinical models. Reuterin, exopolysaccharides, and organic acids modulate the gut microbiota and inhibit pathogenic bacteria; adhesion factors enhance intestinal colonization and participate in barrier repair; histamine, short-chain fatty acids, and membrane vesicles contribute to the suppression of inflammatory pathways and exert immunomodulatory effects. Furthermore, *L. reuteri* regulates the production of GABA, 5-HT, and oxytocin, and modulates the HPA axis, thereby influencing gut–brain axis function and ameliorating visceral hypersensitivity and mood disturbances. These findings indicate that *L. reuteri* holds translational potential in the treatment of IBS. 

However, despite these mechanistic advances, several critical limitations in the current evidence base must be explicitly acknowledged.

The current evidence base is profoundly constrained by strain specificity. The mechanisms discussed in this review are derived from multiple strains—including DSM 17938, ATCC PTA 6475, I5007, D8, LR1, and ZLR003—which differ in their adhesion properties, metabolite production, and immunomodulatory capacities. Functional properties observed in one strain cannot be directly assumed to be present in others, and studies aimed at identifying optimal strains or strain combinations for different IBS subtypes are currently lacking.

In addition, the clinical studies are marked by considerable heterogeneity. For instance, the trial conducted by Cruchet et al., which combined DSM 17938 and ATCC PTA 6475, enrolled adults with moderate-to-severe IBS across all subtypes and employed a 14-week intervention [26], whereas the study by Riezzo et al. examined DSM 17938 exclusively in patients with functional constipation, a population that does not fully represent IBS [55]. Across studies, sample sizes, probiotic doses (typically ranging from 10^8^ to 10^10^ CFU/day), treatment durations, and outcome measures vary substantially, precluding meaningful cross-study comparisons. Inconsistent experimental endpoints, a lack of long-term safety and efficacy data, and the absence of independent replication further widen the translational gap. Moreover, the negative or equivocal findings from several high-quality clinical trials underscore the limitations of the current evidence. For example, multiple randomized controlled trials in children with functional abdominal pain found that, although *L. reuteri* DSM 17938 alleviated symptoms, its effect did not significantly differ from that of placebo [70,71]. In addition, an earlier clinical trial of *L. reuteri* ATCC 55730 in IBS reported higher rates of adverse events such as constipation and bloating in the probiotic group than in the placebo group [72]. Inter-individual variation in gut microbiota composition also profoundly influences probiotic colonization and efficacy. Few studies to date have stratified participants according to baseline gut microbiota profiles, dietary habits, or IBS-associated metabolic signatures, limiting the generalizability of *L. reuteri*-based interventions for clinical translation.

Furthermore, most mechanistic insights originate from animal models and in vitro systems that cannot truly represent the dynamics of IBS. Additionally, there are no published reports on the relationship between post-infectious irritable bowel syndrome and *L. reuteri* intervention. While rodent models of ASD, chemically induced colitis, weaned-piglet intestinal inflammation, and tumor necrosis factor-stimulated mouse intestinal organoids have provided valuable mechanistic validation, the disease states represented in these models are fundamentally distinct from the characteristic features of IBS. Similarly, in vitro experiments often rely on locally high metabolite concentrations and single-cell-type monolayer cultures, conditions that may be difficult to replicate under the physiological environment of the human ileum or colon. These observations collectively indicate that positive findings obtained in animal studies may not necessarily manifest in humans.

Given these limitations, future research should be strategically directed toward several key areas. Well-designed, strain-specific randomized controlled trials are needed that enroll well-phenotyped IBS subgroups, employ standardized dosing regimens and validated outcome measures, and include sufficiently long follow-up periods to assess both durability of effect and safety. Mechanistic validation should be pursued in systems that more closely approximate human physiology, such as patient-derived intestinal organoids, to bridge the gap between animal experiments and clinical reality. In parallel, precision probiotic intervention strategies that integrate individual baseline gut microbiome profiles, host metabolic signatures, and clinical characteristics hold promise for predicting treatment response and enabling personalized *L. reuteri* therapy.

## 8. Conclusions

Different *L. reuteri* strains may contribute to alleviating distinct IBS subtypes through multiple mechanisms, including modulation of the gut microbiota, restoration of barrier integrity, attenuation of inflammation, and regulation of the gut–brain axis. However, current evidence derives predominantly from animal and cell-based studies, and high-quality clinical data remain scarce. The optimal strains, dosages, treatment durations, and combination regimens have yet to be defined, and the development of therapeutics based on active metabolites remains at an early stage.

## Figures and Tables

**Table 1:** Antimicrobial mechanisms of *L. reuteri* metabolites and their target microorganisms.

Metabolite	Targeted Bacterial Strains	Mechanism	References
Reuterin	Gram-positive bacteria, Gram-negative bacteria, yeasts, molds, and protozoa	3-HPA suppresses antioxidant enzyme activity, elevating intracellular ROS levels.	[9,10,11,12]
	*C. difficile*	Disrupt bacterial membrane integrity and enhance antibiotic sensitivity.	[13]
EPS	*Salmonella typhi*	Physical barriers inhibit bacterial attachment and promote Reuterin accumulation.	[14]
SCFAs	*E. coli*	Disrupts the charge balance of cell membranes, affects protein synthesis and enzyme activity, and reduces inflammatory levels.	[15,16,17,18]

Note: The antimicrobial mechanisms listed are general properties of *L. reuteri* metabolites demonstrated in experimental studies. They do not constitute direct clinical evidence for the management of IBS.

## Data Availability

The conclusions presented in this review are based on comprehensive literature synthesis and critical analysis, with no original datasets being collected or analyzed during the preparation of this work.
